# Engineering protein production by rationally choosing a carbon and nitrogen source using *E. coli* BL21 acetate metabolism knockout strains

**DOI:** 10.1186/s12934-019-1202-1

**Published:** 2019-09-04

**Authors:** Gema Lozano Terol, Julia Gallego-Jara, Rosa Alba Sola Martínez, Manuel Cánovas Díaz, Teresa de Diego Puente

**Affiliations:** Department of Biochemistry and Molecular Biology (B) and Immunology, Faculty of Chemistry, University of Murcia, Campus of Espinardo, Regional Campus of International Excellence ‘‘Campus Mare Nostrum’’, P.O. Box 4021, 30100 Murcia, Spain

**Keywords:** Recombinant proteins, Acetate overflow, *Escherichia coli*, Lysine acetylation, GFP, Medium composition

## Abstract

**Background:**

*Escherichia coli* (*E. coli*) is a bacteria that is widely employed in many industries for the production of high interest bio-products such as recombinant proteins. Nevertheless, the use of *E. coli* for recombinant protein production may entail some disadvantages such as acetate overflow. Acetate is accumulated under some culture conditions, involves a decrease in biomass and recombinant protein production, and its metabolism is related to protein lysine acetylation. Thereby, the carbon and nitrogen sources employed are relevant factors in cell host metabolism, and the study of the central metabolism of *E. coli* and its regulation is essential for optimizing the production of biomass and recombinant proteins. In this study, our aim was to find the most favourable conditions for carrying out recombinant protein production in *E. coli* BL21 using two different approaches, namely, manipulation of the culture media composition and the deletion of genes involved in acetate metabolism and Nε-lysine acetylation.

**Results:**

We evaluated protein overexpression in *E. coli* BL21 wt and five mutant strains involved in acetate metabolism (Δ*acs*, Δ*ackA* and Δ*pta*) and lysine acetylation (Δ*patZ* and Δ*cobB*) grown in minimal medium M9 (inorganic ammonium nitrogen source) and in complex TB7 medium (peptide-based nitrogen source) supplemented with glucose (PTS carbon source) or glycerol (non-PTS carbon source). We observed a dependence of recombinant protein production on acetate metabolism and the carbon and nitrogen source employed. The use of complex medium supplemented with glycerol as a carbon source entails an increase in protein production and an efficient use of resources, since is a sub-product of biodiesel synthesis. Furthermore, the deletion of the *ackA* gene results in a fivefold increase in protein production with respect to the wt strain and a reduction in acetate accumulation.

**Conclusion:**

The results showed that the use of diverse carbon and nitrogen sources and acetate metabolism knockout strains can redirect *E. coli* carbon fluxes to different pathways and affect the final yield of the recombinant protein bioprocess. Thereby, we obtained a fivefold increase in protein production and an efficient use of the resources employing the most suitable strain and culture conditions.

## Background

*Escherichia coli* (*E. coli*) is a gram-negative bacteria characterized by its high growth rate, the simplicity of its genome, its easy handling and its capacity to grow in different culture conditions. Therefore, *E. coli* is one of the most studied organisms, and it has been largely employed as a model in biological and biotechnological processes in many industries for the production of drugs, recombinant proteins or other bio-products of high interest [1, 2]. Accordingly, a deeper understanding of the central metabolism, including the regulation of *E. coli,* is essential for optimizing the industrial processes based on the use of this bacteria.

*Escherichia coli* is widely employed as a host cell in the production of recombinant proteins with industrial and pharmaceutical targets [3]. To achieve this aim in the most efficient way, it is important to balance the relationship between cell host metabolism and recombinant protein production. In spite of all the advantages that this bacteria offers, the use of *E. coli* may entail production of by-products such as acetate, which involves a decrease in biomass and recombinant protein production [4, 5]. For this reason, several strategies have been developed to limit acetate accumulation, such as the use of different media and culture conditions [6–8], the employment of genetic engineering to limit the formation and accumulation of this compound, the expression of sRNA or the enhancement of the respiratory activity [9–15]. Although K12 is the most studied *E. coli* strain (K strain), *E. coli* BL21 (B strain) is the most used for recombinant protein production because B strains lack some proteases, achieve higher biomass yields and produces much less acetate than *E. coli* K12, even in the presence of excess glucose [13, 16].

*Escherichia coli* is able to grow using different carbon and nitrogen sources, although it shows preferences for some sources. *E. coli* consumes glucose preferentially over other carbon sources by system regulation and Carbon Catabolite Repression (CCR), which involves a sugar transport system known as phosphoenolpyruvate-phosphotransferase system (PTS system). Glycerol is a non-PTS carbon source, which is expansively employed, since it is obtained as a sub-product of biodiesel synthesis [17]. Regarding nitrogen sources, *E. coli* is capable of using diverse nitrogen compounds to grow but preferentially consumes inorganic ammonium [18]. Moreover, a linkage between carbon and nitrogen sources has been reported through α-ketoglutarate, one of the key intermediates of the TCA cycle, which is required to convert ammonia into glutamate [19, 20].

**Fig. 1:**
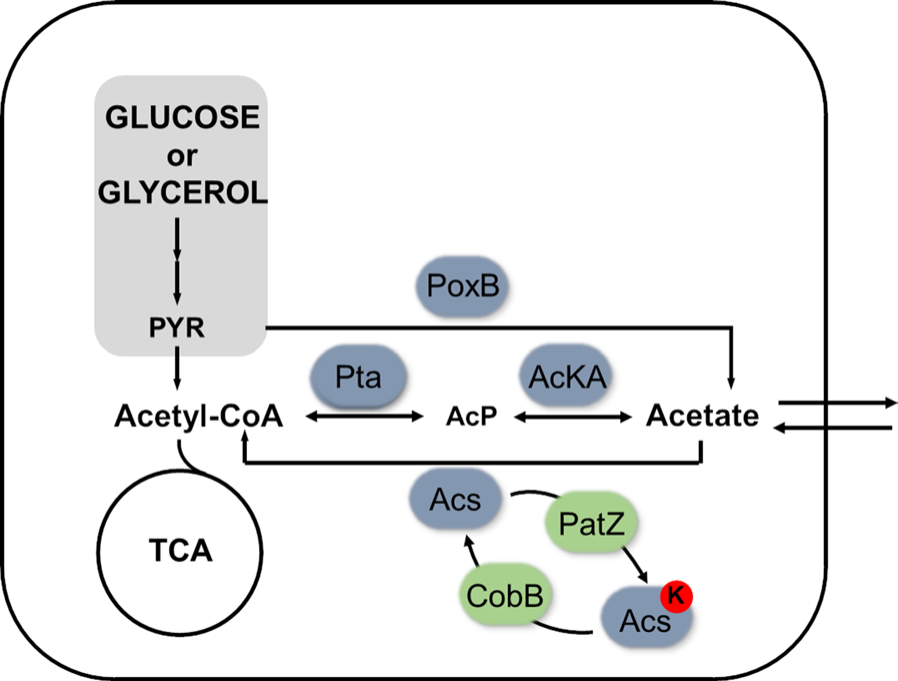
*E. coli* acetate metabolism. Shown in blue are the enzymes responsible for acetate overflow, namely, PoxB, Pta, AckA and Acs. Shown in green are the lysine acetylation enzymes PatZ and CobB. Lysine acetylation of Acs is shown with a red lysine

Acetate metabolism has been largely studied in *E. coli*. This metabolite is excreted and then reincorporated (acetate overflow) in *E. coli* metabolism mainly when glucose is used as the carbon source [5]. Three different pathways catalysed by the enzymes Pta–AckA, Acs and PoxB are responsible for acetate overflow in *E. coli*. The Pta–AckA pathway is formed by the phosphotransacetylase (Pta) and acetate kinase (AckA) enzymes, whose role is the conversion of acetate into acetyl-CoA through an acetyl-phosphate intermediate in a reversible way [21, 22]. Acetyl-CoA synthetase (Acs) catalyses the conversion of acetate into acetyl-CoA through an acetyl-AMP intermediate in an irreversible way. Furthermore, this pathway is a high affinity and well-regulated pathway, whereas the Pta–AckA route is a low affinity pathway [23–25]. Finally, PoxB catalyses an acetate production pathway through pyruvate decarboxylation (Fig. 1).

Nε-lysine acetylation is a post-translational modification, which is linked with acetate metabolism and carbon fluxes through the intermediate metabolites acetyl-P and acetyl-CoA [26, 27]. This modification may alter the activity and conformation of some proteins, inducing its aggregation [28], and it is involved in multiple processes such as cell metabolism, protein–protein interactions, and cell localization [13, 29, 30]. Considering that, its regulation and abundance are relevant factors to take into account in recombinant protein production. Nε-acetylation can occur in a chemical way through acetyl-CoA and acetyl-P metabolites as acetyl donors, or in an enzymatic manner through acetyltransferase and deacetylase enzymes [31]. PatZ is the best characterized acetyltransferase in *E. coli*, and it belongs to the GNATs family [32]. Regarding the reversibility of this process, CobB is the best characterized deacetylase of *E. coli*, and it belongs to the sirtuin deacetylase family [31, 33] (Fig. 1).

The main objective of this study was to determine how acetate metabolism and the use of diverse carbon and nitrogen sources affected overexpression and acetylation levels of recombinant proteins. To reach this target, we employed Green Fluorescent Protein, GFP, as a reporter for monitoring protein overexpression by fluorescence level. In the present study, *E. coli* BL21 wt and five mutant strains involved in acetate metabolism (Δ*acs*, Δ*ackA* and Δ*pta*) and lysine acetylation (Δ*patZ* and Δ*cobB*) were grown aerobically in minimal medium M9 (inorganic ammonium nitrogen source) and in complex TB7 medium (peptide-based nitrogen source) supplemented with glucose (PTS carbon source) or glycerol (non-PTS carbon source). Under these conditions, the growth rate, stoichiometric growth parameters, organic extracellular acids, overexpression levels of recombinant GFP protein and lysine acetylation levels have been evaluated. Thus, in the end, process optimization boils down to finding the appropriate combination of growth conditions and genetic factors that will result in the highest amount of protein.

## Results

### Physiological characterization of the strains overexpressing pRSETA-GFP

To determine the specific growth rates (µ_max_), biomass yields (Y_X/S_) and specific carbon consumption rates (q_s_) for *E. coli* BL21 wt and deficient strains (Δ*patZ*, Δ*cobB*, Δ*acs*, Δ*ackA* and Δ*pta*) overexpressing pRSETA-GFP, all strains were grown in minimal medium M9 or in complex TB7 medium supplemented with 20 mM glucose or 40 mM glycerol. Specific growth rates are shown in Table 1, and the biomass yields and specific carbon consumption rates are provided in Table 2. Cells grown at OD_600_ and acetate, glucose and glycerol extracellular concentrations for all strains are shown in Additional file 1: Figures S1–S6.

**Table 1:** Specific growth rates for *E. coli* BL21strains growing in all culture conditions

	µ_max_ (h^−1^)			
	Glucose TB7	Glycerol TB7	Glucose MM9	Glycerol MM9
wt	0.99 ± 0.09	1.00 ± 0.11	0.45 ± 0.03	0.38 ± 0.02
Δ*patZ*	0.86 ± 0.01	0.74 ± 0.02	0.49 ± 0.01	0.43 ± 0.01
Δ*cobB*	0.78 ± 0.03	0.72 ± 0.01	0.43 ± 0.04	0.32 ± 0.01
Δ*acs*	0.99 ± 0.06	1.05 ± 0.06	0.53 ± 0.03	0.43 ± 0.01
Δ*ackA*	0.62 ± 0.01	0.58 ± 0.02	0.47 ± 0.01	0.40 ± 0.02
Δ*pta*	0.80 ± 0.04	0.44 ± 0.06	0.14 ± 0.01	0.26 ± 0.03

**Table 2:** Stoichiometric parameters, biomass yield (Y_X/S_) and specific carbon consumption rates (q_s_)

	Y_X/S_ (g/mmolC)				q_s_ (mmolC/gh)			
	Glucose TB7^a^	Glycerol TB7^a^	Glucose MM9^a^	Glycerol MM9^a^	Glucose TB7^a^	Glycerol TB7^a^	Glucose MM9^a^	Glycerol MM9^a^
wt	0.013	0.007	0.016	0.009	− 34.49	− 63.34	− 13.20	− 18.91
Δ*patZ*	0.012	0.006	0.013	0.009	− 32.39	− 57.28	− 17.73	− 22.19
Δ*cobB*	0.012	0.006	0.010	0.008	− 30.62	− 52.80	− 19.64	− 17.84
Δ*acs*	0.015	0.008	0.013	0.007	− 31.71	− 60.96	− 18.46	− 27.23
Δ*ackA*	0.020	0.009	0.018	0.006	− 14.64	− 31.08	− 12.36	− 30.96
Δ*pta*	0.018	0.002	0.007	0.006	− 21.03	− 32.62	− 9.75	− 21.31

^a^Each value is the average of three independent experiments with a standard deviation of less than 10%

Several differences in the specific growth rates were observed between culture media. In general, a higher µ_max_ was obtained in TB7 than in MM9, with a 30–60% increase in TB7 depending on the strain, while a lower µ_max_ was obtained using glycerol in comparison to glucose, although in this case, the difference was markedly less pronounced. The highest µ_max_ values in complex medium were observed for the wt and Δ*acs* strains. The mutants associated with enzymatic lysine acetylation modification, Δ*patZ* and Δ*cobB*, showed a similar profile to wt, but a lower µ_max_ value than that in TB7 medium. We found great differences between wt and the gene deletion mutant strains Δ*ackA* and Δ*pta*. In minimal medium, the specific growth rate of the Δ*ackA* strain was similar to the wt strain, whereas in TB7 medium, this value decreased by 39% with respect to the wt value. The deletion of *pta* severely affected mutant strain growth in all media conditions, and µ_max_ was reduced by 40–70% when compared to wt, except in glucose TB7 medium, which was similar to wt (Table 1).

Attending to biomass yield, Y_X/S_ was higher in media supplemented with glucose than with glycerol, and no differences were found between complex or minimal media for all the strains. All strains showed a similar behaviour with respect to wt, except the related acetate metabolism mutants, Δ*ackA* and Δ*pta*. We observed the lowest Y_X/S_ for the *pta* gene deletion strain, except in glucose-TB7 medium, where the Δ*pta* biomass yield was higher than wt, whereas the Δ*ackA* strain showed the highest biomass yield in all culture conditions except for the glycerol-MM9 medium. With regard to the specific carbon consumption rate, we observed higher values in glycerol than in glucose and in TB7 than in MM9. It should be noted that we observed differences in Δ*pta* and Δ*ackA* with respect to the rest of the strains, since Δ*ackA* and Δ*pta* showed the lowest q_s_ values in complex media and minimal media, respectively (Table 2).

### Organics acid analysis

**Fig. 2:**
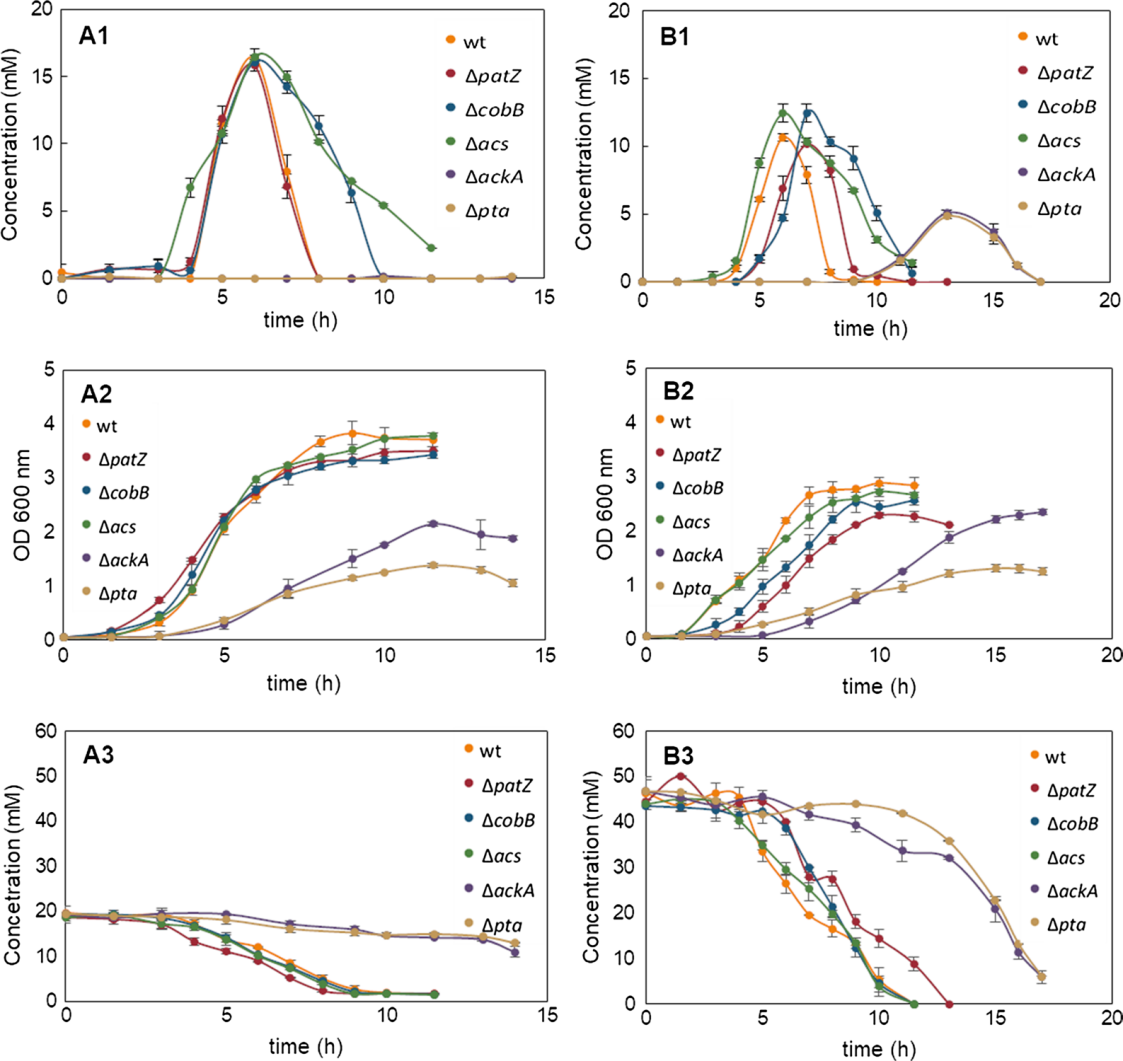
Extracellular acetate, glucose and glycerol concentrations, and cell grown at OD_600_. Extracellular acetate concentrations detected for *E. coli* BL21 wt, Δ*patZ*, Δ*cobB*, Δ*acs*, Δ*ackA* and Δ*pta* growing in glucose-TB7 (**a1**) and glycerol-TB7 (**b1**). Extracellular glucose (**a2**) and glycerol (**b2**) concentrations, and cell grown in glucose-TB7 (**a3**) or glycerol-TB7 (**b3**) for *E. coli* BL21 wt, Δ*patZ*, Δ*cobB*, Δ*acs*, Δ*ackA* and Δ*pta*

Extracellular concentrations of organic acids were evaluated for *E. coli* BL21 wt and deletion strains (Δ*patZ*, Δ*cobB*, Δ*acs*, Δ*ackA* and Δ*pta*) overexpressing pRSETA-GFP in minimal M9 medium or in complex TB7 medium supplemented with glucose or glycerol as the carbon source. Acetate, pyruvate, lactate, formate, citrate and α-ketoglutarate concentrations were analysed, but only extracellular acetate concentrations were detected. The extracellular acetate concentrations are shown in Fig. 2 and specific acetate production and consumption rates in Table 3.

**Table 3:** Specific acetate production and consumption rates

	Acetate production rate (mmol/gh)				Acetate consumption rate (mmol/gh)			
	Glucose TB7^a^	Glycerol TB7^a^	Glucose MM9^a^	Glycerol MM9^a^	Glucose TB7^a^	Glycerol TB7^a^	Glucose MM9^a^	Glycerol MM9^a^
wt	13.62	7.80	0.35	0.14	− 7.02	− 5.16	−0.11	−0.11
Δ*patZ*	9.79	13.90	0.27	0.16	− 6.85	− 6.80	−0.13	−0.07
Δ*cobB*	16.49	11.10	0.54	0.38	− 1.70	− 2.04	−0.20	−0.08
Δ*acs*	11.06	10.54	0.38	0.18	− 1.70	− 2.29	−0.20	−0.02
Δ*ackA*	0.12	2.79	0.09	0.11	−0.21	− 1.87	−0.11	−0.05
Δ*pta*	0.17	3.64	0.08	0.07	−0.20	− 1.41	−0.06	−0.06

^a^Each value is the average of three independent experiments with a standard deviation of less than 10%

**Fig. 3:**
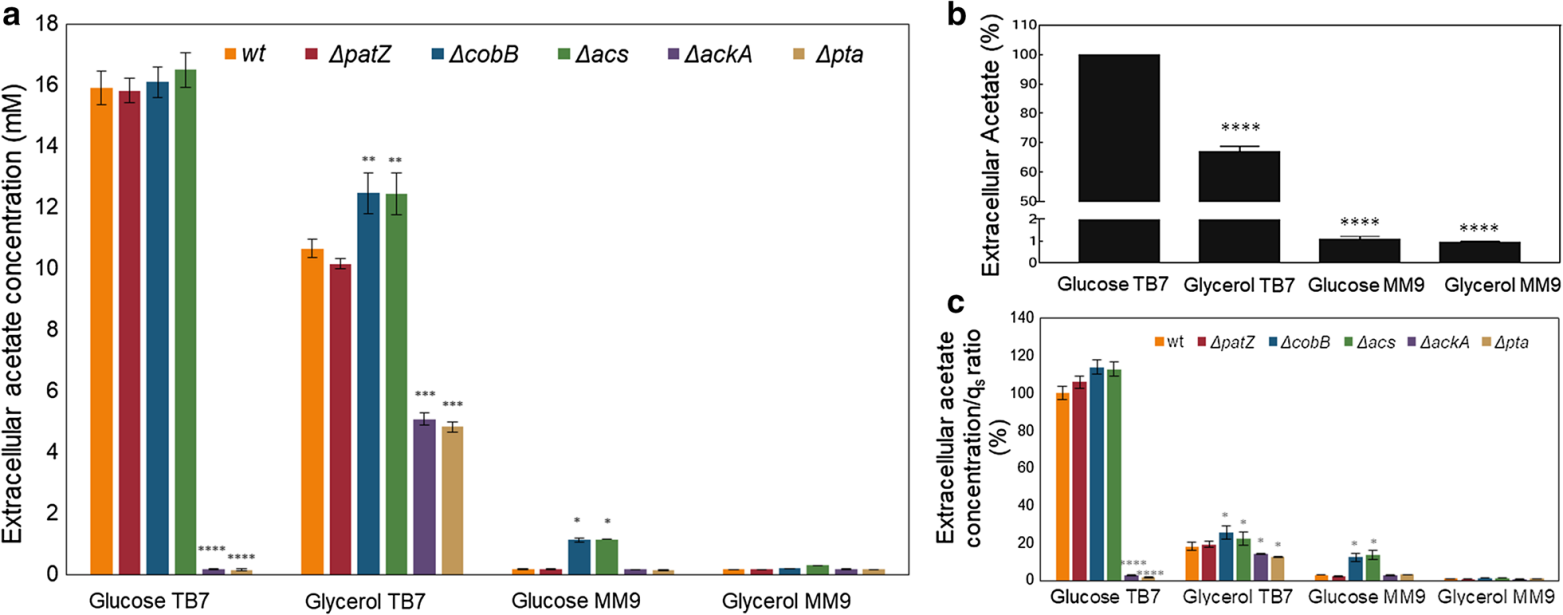
Maximal extracellular acetate concentrations. **a** Maximal acetate concentrations detected for *E. coli* BL21 wt and knockout strains growing in MM9 or TB7 medium supplemented with glucose or glycerol as the carbon source. **b** Acetate extracellular concentration percentage relative to *E. coli* BL21 wt growing on glucose-TB7. **c** Extracellular acetate concentrations/q_s_ ratio expressed as a percentage relative to *E. coli* BL21 wt growing on glucose-TB7. Statistical tests, including two-way ANOVA (**a** and **c**) and one-way ANOVA (**b**), were carried out in order to evaluate differential significance between knockout mutants and the wt strain in each condition (**a** and **c**), and the differential significance between glucose-TB7 and the other media in the wt strain (**b**) (p-value < 0.0001 (****), < 0.001 (***), < 0.01 (**), < 0.05(*))

Extracellular acetate was accumulated during the exponential growth phase and then reincorporated in mid-exponential phase for *E. coli* BL21 wt. In contrast, acetate was incorporated just at the entry into the stationary growth phase in Δ*cobB* and Δ*acs*. In particular, acetate reincorporation in the *cobB* and *acs* gene deletion strains was slowed down in all culture conditions (Fig. 2). These mutants showed a 2.7- and 2.5-fold decrease in the acetate consumption rate relative to the wt in both carbon sources in complex medium, respectively (Table 3). Acetate production and consumption rates were higher in glucose supplemented medium than in glycerol, and specifically, the acetate production rate was not significantly different between strains, with the exception of the Δ*ackA* and Δ*pta* strains. These mutants showed about a 2.7-fold decrease in both the acetate production rate and the consumption rate with respect to wt in glycerol-TB7, and in contrast, no extracellular acetate was detected in glucose-TB7 medium. These mutants also showed the lowest values of acetate consumption rate. Acetate excretion in minimal medium was quantitatively insignificant when compared with TB7 medium, as seen in Additional file 1: Figures S1–S6 and Table 3. Maximal acetate concentrations detected in each condition and strain are shown in Fig. 3a, the acetate extracellular concentration normalized to the maximum value obtained for *E. coli* BL21 wt is shown in Fig. 3b, and extracellular acetate concentrations/q_s_ ratio expressed as a percentage relative to *E. coli* BL21 wt growing on glucose-TB7 is shown in Fig. 3c.

Several differences were observed in acetate extracellular concentrations depending on the culture media and the strains studied. For *E. coli* wt, we found an elevated concentration of extracellular acetate when grown in complex media, from 10.6 to 16 mM depending on the carbon source, and a low acetate concentration when grown in minimal medium, approximately 0.16 mM or less (Fig. 3a). We noticed that the acetate concentration was dependent on the carbon source used, as clearly seen in Fig. 3b, and extracellular acetate concentrations/q_s_ ratio showed a profile similar to that for maximal acetate concentrations Fig. 3b. Regarding the use of glycerol, we also found a decrease in acetate concentration by more than 30% with respect to glucose, which was more pronounced when growing in complex media (Fig. 3).

In the same way, we observed that extracellular acetate concentrations were altered in the mutants related to acetate metabolism and in the mutants associated with lysine acetylation modifications. The Δ*acs* and Δ*cobB* mutants showed the highest acetate values in all conditions tested, especially in glucose minimal medium (approximately 1 mM), while the other mutants showed a negligible acetate concentration (approximately 0.16 mM). Concerning *ackA* and *pta* gene deletion strains, almost imperceptible amounts of acetate were observed in all media, except in glycerol-TB7, although to a lesser extent than the other strains in the same media (Fig. 3a).

**Fig. 4:**
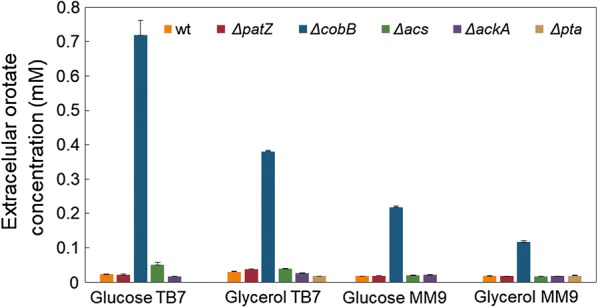
Maximal extracellular orotate concentrations. Maximal extracellular orotate concentrations detected for *E. coli* BL21 wt and knockout strains growing in MM9 or TB7 medium supplemented with glucose or glycerol as the carbon source

Whereas we were studying extracellular organic acids, we observed a compound with absorption at 280 nm. When we analysed this compound by LC–MS/MS, we noticed that it was orotic acid. Orotate is a pyrimidine pathway intermediate that can be excreted during growth, and this could lead to carbon wastage and less biomass and macromolecule production [34], so orotate could affect recombinant protein production. Orotate was excreted during the exponential growth phase and was not totally incorporated (Additional file 1: Figure S7). Orotate maximal extracellular concentrations are shown in Fig. 4.

In general, we observed that orotate excretion was almost imperceptible in all strains and conditions (approximately 0.02 mM), without huge differences, with the exception of the Δ*acs*, Δ*cobB* and Δ*pta* strains. No excretion of orotate was observed in the Δ*pta* strain growing with glucose as a carbon source. The Δ*acs* strain showed differences in comparison to the wt in TB7, showing concentrations greater than 40–60% with respect to the wt in this medium. The strain with the greatest orotate excretion was Δ*cobB* (0.8–0.12 mM), with up to a 35-fold increase with respect to wt growing on glucose-TB7. Furthermore, the orotate concentration for the Δ*cobB* strain was higher in TB7 than MM9 and higher in glucose than glycerol (Fig. 4).

### Relative transcription level determined by qRT-PCR

The imbalance between carbon source catabolism and respiratory metabolism in *E. coli* involves the accumulation of acetyl-CoA, and consequently, the acetate overflow. Carbon catabolite repression and regulation of central carbon metabolism play a relevant role in carbon metabolism balance. Under these conditions the predominant regulators are the cAMP receptor protein (Crp), the catabolite repressor activator (Cra) and the aerobic respiration control protein (ArcA) [5].

**Fig. 5:**
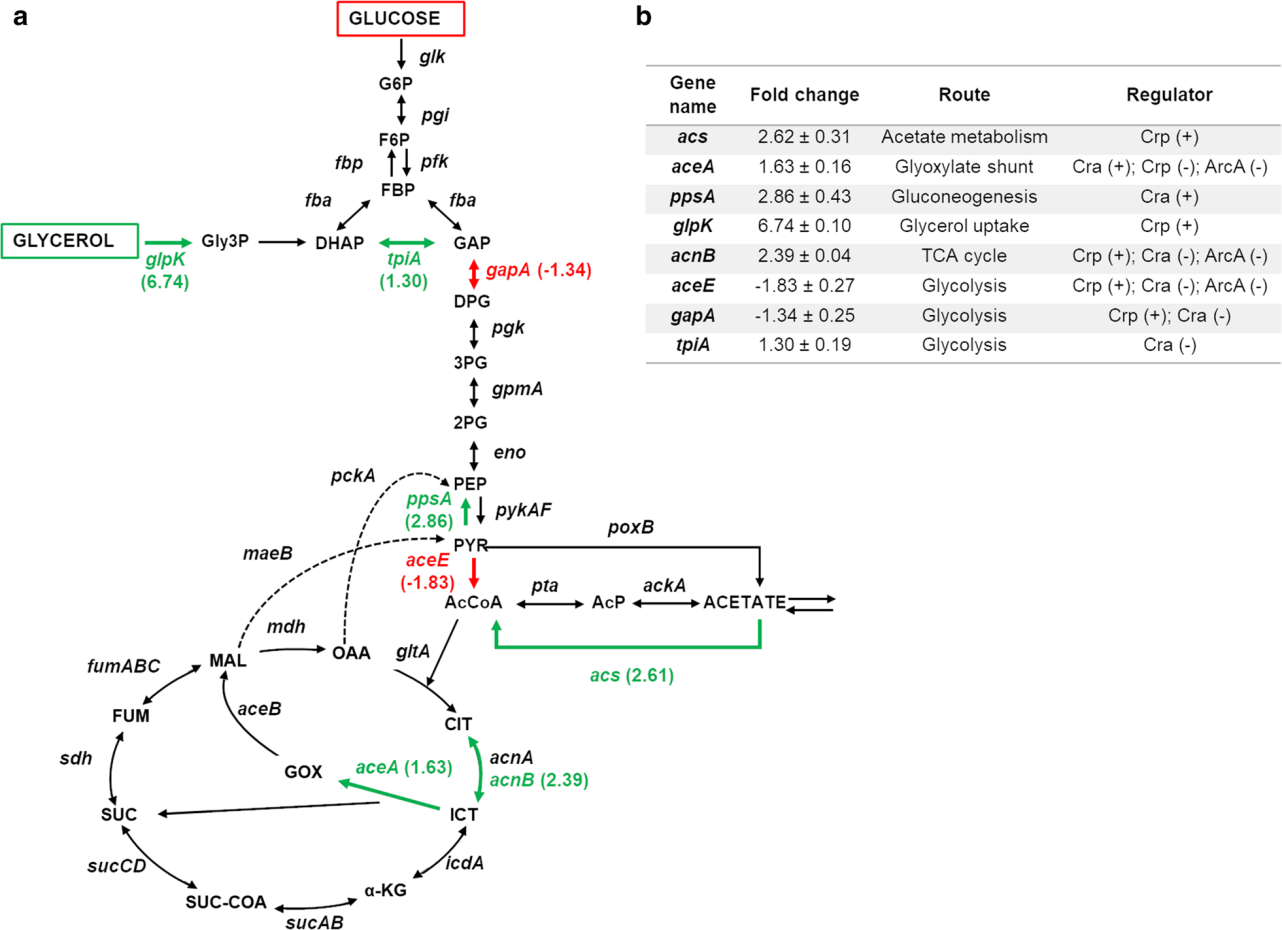
Relative gene transcription values in *E. coli* BL21 wt. Relative gene transcription of *E. coli* BL21 wt growing in glycerol, as compared to *E. coli* Bl21 wt growing in glucose. **a** The genes of the central metabolism studied are show in red (underexpressed in glycerol with respect to glucose) or in green (overexpressed in glycerol with respect to glucose). Metabolite abbreviations: *Gly3P* glycerol-3-phosphate, *G6P* glucose-6-phosphate; *F6P* fructose-6-phosphate, *FBP* fructose-1,6-biphosphate, *DHAP* dihydroxyacetone phosphate, *GAP* glyceraldehyde-3-phosphate, *DPG* 1,3-bisphosphoglycerate, *3PG* 3-phosphoglycerate, *2PG* 2-phophoglycerate, *PEP* phosphoenolpyruvate, *PYR* pyruvate, *AcCoA* acetyl coenzyme A, *AcP* acetyl phosphate, *CIT* citrate, *ICT* isocitrate, *GOX* glyoxylate, *α-KG* α-ketoglutarate, *SUC-CoA* succinyl-coenzyme A, *SUC* succinate, *FUM* fumarate, *MAL* malate, *OAA* oxaloacetate. **b** The table shows fold changes of transcription level of selected genes in glycerol growth compared with glucose growth, the route to which they belong and the global regulators that regulates positively (+) or negatively (−) its expression

A qRT-PCR assay had been carried out in order to analyse the differences in the activity of these global regulators and its relation with the metabolism changes and acetate overflow, when *E. coli* BL21 grows in different carbon sources. The genes analysed (*acs*, *aceA*, *ppsA*, *glpK*, *acnB*, *aceE*, *gapA* and *tpiA*) are regulated by these transcription factors and belongs to the different pathways of the central carbon metabolism (Fig. 5). Relative gene transcription (fold change) was based on the expression levels of *E. coli* BL21 wt growing in glycerol versus glucose employing a defined medium (Fig. 5).

As expected, *glpK* (glycerol kinase gene) showed the higher overexpression of all selected genes in glycerol growth. The expression of this gene is activated by cAMP-Crp [35], and GlpK catalyses the rate-limiting step in glycerol utilization [36]. As regards to the genes studied belonging to glycolysis, *tpiA* (triose phosphate-isomerase gene) was overexpressed in glycerol and *gapA* (glyceraldehyde-3-phosphate dehydrogenase gene) was overexpressed in glucose growth, but the change in the expression was not deeply affected. The expression of both genes is repressed by Cra, and *gapA* expression is activated by cAMP-Crp [37]. *aceE* (pyruvate dehydrogenase gene) was underexpressed in *E. coli* BL21 grown in glycerol, and its expression is activated by cAMP-Crp and repressed by Cra and ArcA [37]. Regarding to acetate metabolism, *acs* (acetyl-CoA synthetase gene) expression was upregulated in *E. coli* BL21 grown in glycerol and it is activated by cAMP-Crp [37]. In the TCA cycle, *acnB* (aconitase B gene) was overexpressed in glycerol growth and it is positively regulated by cAMP-Crp and repressed by Cra and ArcA [35, 37]. As regards to glyoxylate shunt we studied the relative gene transcription value of *aceA* (isocitrate lyase gene), whose expression was downregulated in *E. coli* BL21 grown in glycerol. *aceA* expression is activated by Cra and repressed by ArcA and cAMP-Crp [38, 39]. Finally, *ppsA* (phosphoenolpyruvate synthetase gene) expression was upregulated in glycerol growth, with a similar value than *acs* and *acnB*, and it is activated by Cra [38] (Fig. 5).

### Overexpression of GFP protein

To evaluate recombinant protein production in *E. coli* BL21 and mutant derivative strains, we employed Green Fluorescent Protein expression levels (GFP). *E. coli* BL21 wt and its mutant derivative cells carrying pRSETA-GFP were grown in glycerol-TB7, and after 24 h, equal amounts of *E. coli* cells were plated onto LB agar, and fluorescent images of *E. coli* BL21 wt and mutant derivatives expressing GFP were obtained (Additional file 1: Figure S8). It has been demonstrated that the fluorescence of GFP can only be emitted when the protein has the correct tertiary structure [40, 41]. Assuming that GFP fluorescence is an indicator of the correct tertiary folding of GFP, the highest functional GFP production vs fluorescence was detected for the Δ*ackA* strain followed by Δ*cobB* and Δ*pta*.

**Fig. 6:**
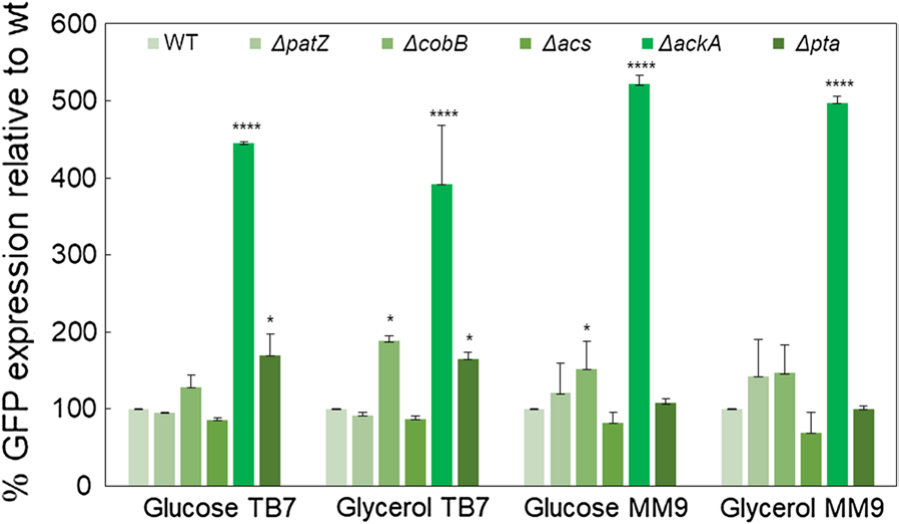
Percentage of GFP expression. Percentage of GFP expression relative to wild-type for *E. coli* BL21 wt and deletion strains in each growth medium. Statistical testing involving two-way ANOVA was carried out in order to evaluate differential significance between knockout mutants and the wt strain in each condition (p-value < 0.0001 (****), < 0.05(*))

Fluorescence intensities were recorded during culture growth, and the highest level of GFP expression was observed when bacterial cells reached the stationary phase (Additional file 1: Figure S9 corresponding to wt). The effects of mutations in the acetate excretion/assimilation and lysine acetylation pathways on maximum GFP expression levels (relative to wild-type) for each strain in different culture media are shown in Fig. 6.

Protein overexpression profiles followed a similar pattern in all culture conditions. The highest GFP expression was noticed for the Δ*ackA* strain (*p* value < 0.0001) in all culture media increasing 4- to 5-fold with respect to the wt. The Δ*acs* strain showed the lowest GFP expression level, although in this case, no significant differences were observed with respect to the wt strain. The Δ*pta* mutant showed 1.6-fold higher GFP expression levels than the wt value in TB7, with a p-value < 0.05, while there was no significant difference between this mutant and the wt in minimal medium. The Δ*cobB* strain also showed an increase in the GFP expression level in comparison to the wt in all culture media, being significantly superior to the wt in glycerol-TB7 and glucose-MM9 (p-value < 0.05) (Fig. 6).

**Fig. 7:**
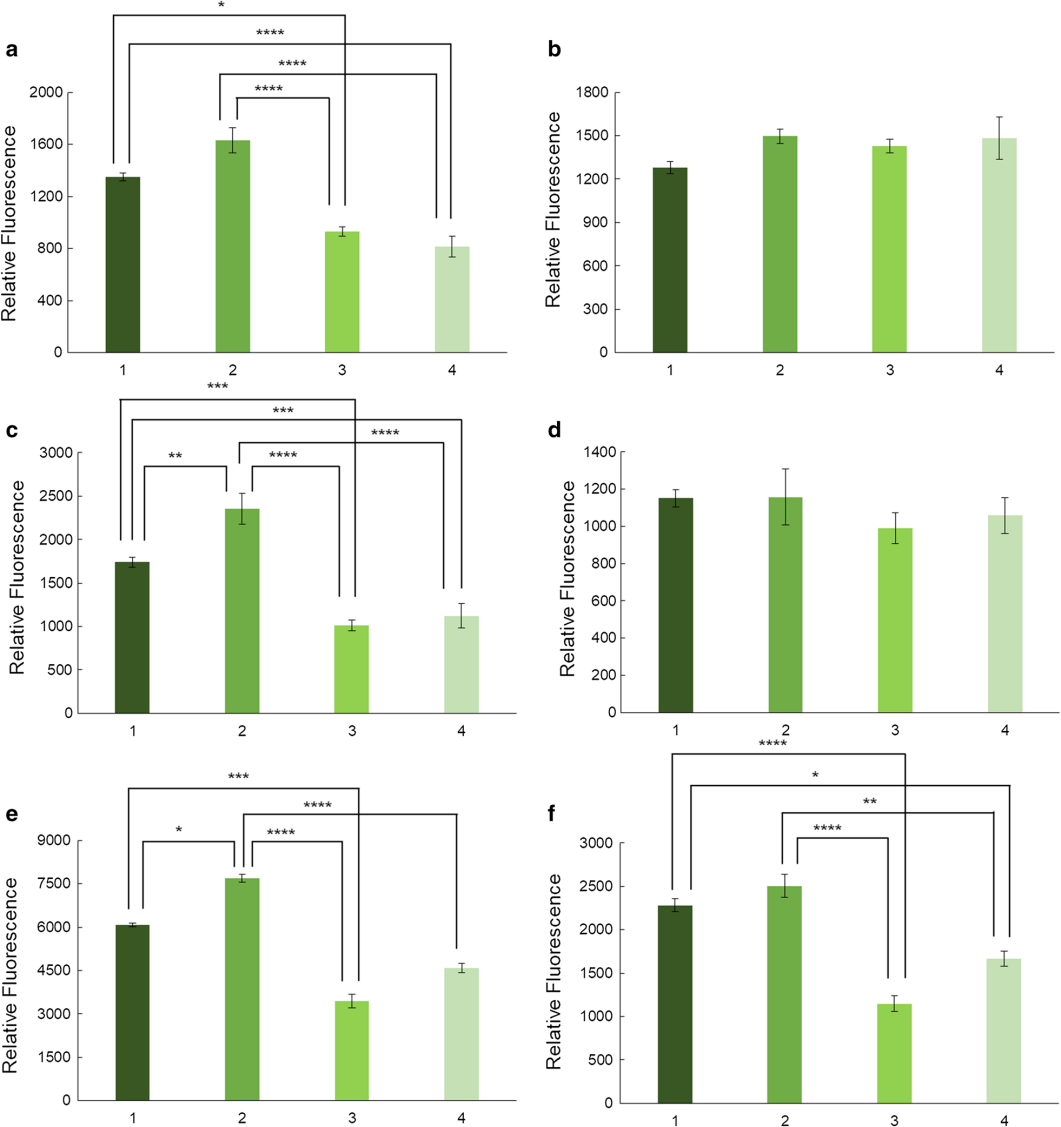
Relative fluorescence intensity for each strain growing in all culture conditions. *E. coli* BL21 wt (**a**) and deletion strains (Δ*patZ* (**b**), Δ*cobB* (**c**), Δ*acs* (**d**), Δ*ackA* (**e**) and Δ*pta* (**f**)). Glucose-TB7 [1] glycerol-TB7 [2], glucose-MM9 [3] and glycerol-MM9 [4]. Statistical analysis by two-way ANOVA was carried out comparing relative fluorescence between culture media in each strain (p-value < 0.0001 (****), < 0.001 (***), < 0.01 (**), < 0.05(*))

To clarify the effect of different media compositions on GFP expression levels, Fig. 6 was broken up into separate figures for each strain and the GFP expression level was indicated as fluorescence intensity normalized to OD_600_ values (Fig. 7).

*Escherichia coli* wt showed higher GFP expression levels in complex than in minimal media (Fig. 7). However, we did not observe significant differences when carbon sources were compared. In contrast, the Δ*cobB* and Δ*ackA* strains showed statistically significant differences between all culture conditions with the greatest GFP expression in glycerol-TB7. The other strains showed the same pattern with the highest expression level supported by glycerol-TB7 growth medium.

### GFP acetylation level analysis

**Fig. 8:**
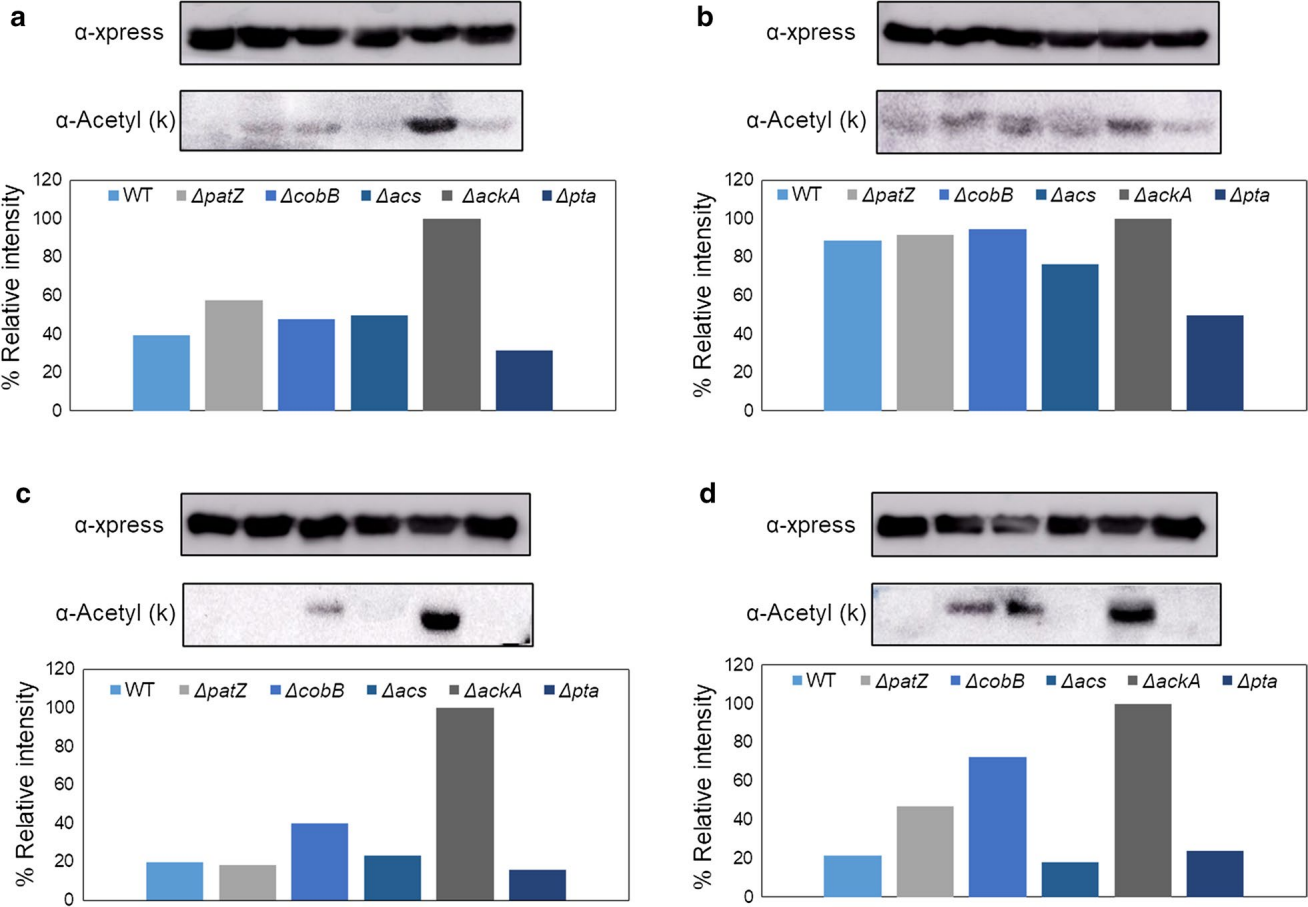
Anti-acetyl lysine and anti-xpress (loading control) western blots. Anti-acetyl lysine and anti-xpress (loading control) western blots of all strains in different culture media including glucose-TB7 (**a**), glycerol-TB7 (**b**), glucose-MM9 (**c**) and glycerol-MM9 (**d**). The graphics represent the relative intensity of the anti-acetyl lysine western blot lanes studied by densitometry. Western blot membranes have the following order: wt (1st lane), Δ*patZ* (2nd lane), Δ*cobB* (3rd lane), Δ*acs* (4th lane), Δ*ackA* (5th lane) and Δ*pta* (6th lane)

Western blot anti-acetyl lysine analysis was carried out to study the GFP acetylation level for the different culture conditions in *E. coli* BL21 wt and deficient strains. We performed two types of immunoblots with two different approaches using two antibodies: anti-xpress (loading control) and anti-acetyl lysine antibody (acetylation level). Thus, we compared the acetylation level of purified GFP between culture media in the same strain. However, we did not observe significant differences between acetylation levels (data not shown). On the other hand, we carried out a comparison of the purified GFP acetylation levels between the strains in the same culture medium (Fig. 8). To evaluate the differences in western blot, we carried out a densitometric analysis of the western blot assays.

The same acetylation pattern was detected for all conditions, with the highest acetylation level corresponding to the Δ*ackA* strain. Moreover, less difference between the acetylation levels was observed in complex than in minimal medium, and in glycerol than in glucose. Concerning each culture medium, in glucose-TB7, we observed an increase (approximately 60%) in the acetylation level of GFP in the Δ*ackA* strain with respect to the wt. In glycerol-TB7, almost no differences in the acetylation level of GFP were found, except for GFP from Δ*pta*, which showed a twofold decrease in acetylation level when compared to wt. In minimal media with both carbon sources (glucose and glycerol), GFP exhibited a similar profile of acetylation, with the most elevated acetylation level corresponding to GFP purified from Δ*ackA* (about 80% more than GFP from wt). For these conditions, GFP from Δ*cobB* also showed a higher acetylation level than wt (Fig. 8).

At the same time, we evaluated the global state of purified proteins from all culture conditions in *E. coli* BL21 wt and deficient strains by analysis of the GFP fluorescence spectrum. Under all conditions tested, GFP showed the same fluorescence spectra with maximum fluorescence emission at 509 nm, which could indicate the same native conformation of purified GFP from all cultures and strains employed, and no relationship between the lysine acetylation level and native protein tertiary structure was noted.

## Discussion

Several bacteria have been explored as hosts for recombinant protein production; however, *E. coli* has been widely used as a workhorse in the production of recombinant proteins of industrial or therapeutic interest due to the simplicity and low cost of its employment, despite some disadvantages that the utilization of *E. coli* imposes [1–3, 42]. Among these disadvantages, one of the biggest is acetate overflow, which is produced in high density cultures in complex and rich media [5, 43]. Furthermore, the study of central metabolism of this bacteria and carbon fluxes is important for the production of biomass and macromolecules, such as recombinant proteins.

In this study, we evaluated different factors that are relevant to the production of recombinant proteins, such as the carbon and nitrogen sources and the accumulation of extracellular acetate by using metabolic engineering to delete acetate pathway genes. Our last aim was to find the most favourable conditions for overexpressing recombinant proteins in *E. coli* BL21.

In MM9 cultures, the carbon source was consumed completely during the exponential growth phase and the cultures reached stationary phase when the glucose or glycerol was depleted (Additional file 1: Figures S1–S6). However, in TB7 cultures, the carbon source was not consumed completely during the exponential growth phase. This behaviour was previously reported by Wolfe et al. [27], who suggested that peptide-based complex media were magnesium limited for bacterial growth. In these cultures, when both acetate and glucose/glycerol were present, *E. coli* BL21 and derivative mutants were able to use both carbon sources simultaneously [13, 44].

Concerning the growth parameters, µ_max_ was higher in complex than in minimal medium and higher in glucose-supplemented culture media than with glycerol (Table 1). In addition, we observed a relationship between the nitrogen source employed and the consumption of the carbon source on the growth rate, with the type of nitrogen source having a greater effect than the carbon source. Thus, the differences between the µ_max_ values on both carbon sources were markedly more pronounced in MM9 than in TB7, over a range of 10 to 20% (Table 1). This result indicated that glucose was a better carbon source in a culture medium based on inorganic ammonium than one based on peptides. This fact was also observed by Bren et al. [20], who suggested that *E. coli* cells grow more slowly on glucose when a single amino acid is the nitrogen source due to high levels of TCA intermediates and low cAMP levels. In contrast, the specific rate of substrate consumption was substantially affected by both nitrogen and carbon sources, whereas the biomass yield was more affected by carbon source, increasing twofold more with glucose than with glycerol.

Acetate is an *E. coli* by-product related to growth and carbon source consumption. This metabolite is a big drawback in biotechnology since concentrations above 40 mM can interfere with recombinant protein production [45]. Aerobic acetate production was maximized in TB7 cultures, while it was practically undetected in minimal medium, and the acetate extracellular concentration increased 1.5-fold more with glucose than with glycerol in *E. coli* BL21 wt (Figs. 2 and 3). This fact can be explained by the imbalance between glucose uptake and the demands for both biosynthesis and energy production [43]. Furthermore, acetate formation during rapid growth in rich medium is additionally aggravated through the availability of free amino acids [37]. Moreover, in this study, deletion of *ackA* and *pta* genes involved a negligible acetate accumulation in glucose-TB7 and a 4.5-fold less in glycerol-TB7 with respect to wt strain. In the case of Δ*ackA* strain only µ_max_ is reduced by 39% with respect to wt strain (Tables 1 and 2 and Fig. 3). Other studies have employed this strategy to reduce acetate production with similar results. Phue et al. [46] observed a twofold decrease in acetate accumulation and a lower decrease in µ_max_ and Y_X/S_ in *E. coli* BL21 Δ*pta*–*ackA* double mutant. Other works showed the same profile for the double mutant [47] and a 14-fold less acetate production in a *pta* knockout strain [22]. Engineering central carbon metabolism pathways is another strategy employed to prevent acetate overflow metabolism, hence the overexpression of Mlc (regulator of PTS system) involved a twofold decrease in acetate production, and no differences in µ_max_ and Y_X/S_ values as compared with wt strain [48]. Whereas the double deletion of *pkyF* and *pkyA* (pyruvate kinase genes) entailed a decrease in acetate production and µ_max_ with respect to wt strain [49]. Furthermore, several studies have focused their efforts in the engineering of metabolism regulators such as IclR and ArcA, which repressed glyoxylate shunt and TCA cycle. When *iclR* regulator is deleted a reduction by twofold in Y_X/S_ and acetate production is observed [50]. Moreover the double deletion strain *iclR*-*arcA* entailed a 70% reduction in acetate production, an increase in biomass yield and a 38% decrease in µ_max_ as compared with wt [51]. Finally a novel strategy showed a decrease by 50–90% in acetate production without changes in µ_max_ and Y_X/S_, through the enhancement of the respiratory activity by the expression of the *Vitreoscilla stercoraria* hemoglobin (VHb) [15]. Therefore the decrease in acetate concentration observed in our study is one of the biggest, and in *ackA* deletion strain only the µ_max_ was affected, moreover the great production of recombinant protein supported by this mutant revealed a negligible loss of carbon (Figs. 3 and 6).

The relative transcription level differences of genes regulated by cAMP-Crp, Cra and ArcA were related with the activity of these transcription factors and with the differences observed in metabolism when *E. coli* BL21 grown in glucose or in glycerol as carbon sources. As regards to ArcA, this regulator repress the expression of TCA cycle and glyoxylate shunt genes [37], however we observed an overexpression of *acnB* and *aceA* genes in glycerol growth (Fig. 5). During rapid growth in glycerol a repressing influence of Arca on the TCA cycle genes might be outcompeted by an activating influence of cAMP-Crp [37]. Furthermore, Crp is activated by its union to cAMP in absence of a PTS carbon source and it activates the transcription of genes of the lower glycolytic pathway, the TCA cycle, the pyruvate dehydrogenase complex and genes for non-PTS carbon source utilization [37, 52]. Additionally Cra is repressed by fructose-1,6-bisphosphate (FBP) and causes strongly repression on the glycolytic pathway genes and the pyruvate dehydrogenase complex and it activates the transcription of gluconeogenic and glyoxylate shunt genes [37, 53]. Our results were in accordance with higher activities of both, cAMP-Crp and Cra regulators, under glycerol conditions as compared to glucose growth, since we observed an overexpression in genes from TCA cycle, acetate metabolism, glycerol utilization, gluconeogenesis and glyoxylate shunt, whereas genes from glycolytic pathway and pyruvate dehydrogenase were underexpressed (Fig. 5). Moreover, our results would suggest that an increment in the flux through TCA cycle, glyoxylate shunt, acetate consumption and gluconeogenesis is produced when *E. coli* BL21 grown in glycerol as the sole carbon source, which could explain a less carbon lost and acetate overflow under these conditions. Nevertheless, acetyl-CoA formation would be favoured under glucose growth, hence it could be promote acetate overflow. All these data showed the relevance of the correct regulation of central metabolism, and the benefit on the use of glycerol, a non-PTS carbon source, which would allow carbon scavenging and less carbon lost. Martínez-Gómez et al. [35] analysed the relative transcription level in *E. coli* K12 JM101 strain grown in glycerol with respect to *E. coli* K12 JM101 strain grown in glucose. They showed the same expression profile than the data obtained in our study, although it is known that both strains have distinctive differences in genotypic and phenotypic attributes [54]. All the foregoing reasons reflected the competitive regulation of central metabolic pathways through the global transcriptional regulators Cra, Crp, and ArcA.

**Fig. 9:**
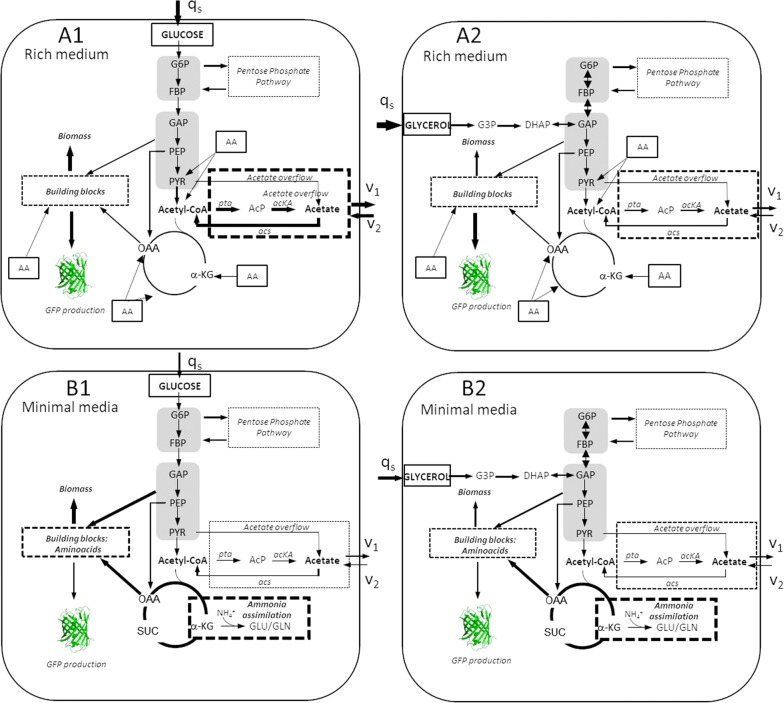
Central metabolic pathway of *E. coli* BL21 at different aerobic growth conditions. A. Rich medium supplemented with glucose (**a1**) or glycerol (**a2**). B Minimal medium supplemented with glucose (**b1**) or glycerol (**b2**). The thickness of the arrows represents the relative flux comparing growth cultures. (q_s_, data shown in Table 2; v_1_, acetate production rate and v_2_, acetate consumption rate, data shown in Table 3)

The production of recombinant proteins was evaluated by monitoring the fluorescence of the reporter protein GFP in the different culture media employed (Figs. 5, 6, 7). Protein expression increased twofold more in TB7 than in MM9 (Fig. 7). This higher protein production in TB7 could be due to the elevated growth rate and biomass yield achieved in this media (Tables 1 and 2), as it has been published that recombinant protein production is higher in cultures growing at a high rate, despite elevated acetate concentrations [55]. Furthermore, in other work where the influence of rich and defined media over *E. coli* BL21 was evaluated, it is shown that a large fraction of resources are directed to synthesizing the enzymes required for amino acid synthesis during growth in a mineral salt medium, while these resources can be redirected to other tasks in cells growing rapidly in rich medium (Fig. 9). A higher expression of proteins and enzymes involved in protein synthesis and folding was detected in rich medium than in defined medium [37], which suggests a possible elevated protein overexpression in rich TB7 medium than in defined MM9. With respect to the carbon source, protein expression levels were higher when cultures were supplemented with glycerol (20 to 30% more than in glucose-TB7) (Fig. 7). This elevated production in glycerol could be due to the lower production of acetate with respect to glucose (Fig. 3). Figure 9 shows a simplified model for the central metabolic pathway of *E. coli* BL21 at different aerobic growth conditions employed in this study. The thickness of the arrows represents the flow belonging to the pathways at these growth conditions and related to other culture conditions, attending to the qRT-PCR, q_s_ and acetate production/consumption rates data (Tables 2, 3 and Fig. 5). Our results revealed that the optimal growth medium for GFP production was rich media supplemented with glycerol using *E. coli* BL21 wt (Fig. 9a2). However, the effect of the carbon source on the inflow of GFP production was less important than that of the nitrogen source. Furthermore, gene deletion had a significant impact on protein production.

The Δ*patZ* strain showed a physiological behaviour similar to the wt strain. GFP overexpression and acetylation levels were not affected by *patZ* deletion (Tables 1 and 2 and Figs. 2, 3, 4, 5, 6, 7, 8). These results reveal that this acetyltransferase has only minor effects on global acetylation [56] since abundant lysine acetylation in *E. coli* results from the build-up of metabolic intermediates, mainly acetyl-P, under conditions that favour acetate by non-enzymatic acetylation [57, 58]. Moreover, 4 new acetyltransferases have been identified recently in *E. coli*, RimI, YiaC, YjaB, and PhnO [59], which could catalyse enzymatic acetylation of proteins in the absence of PatZ.

**Fig. 10:**
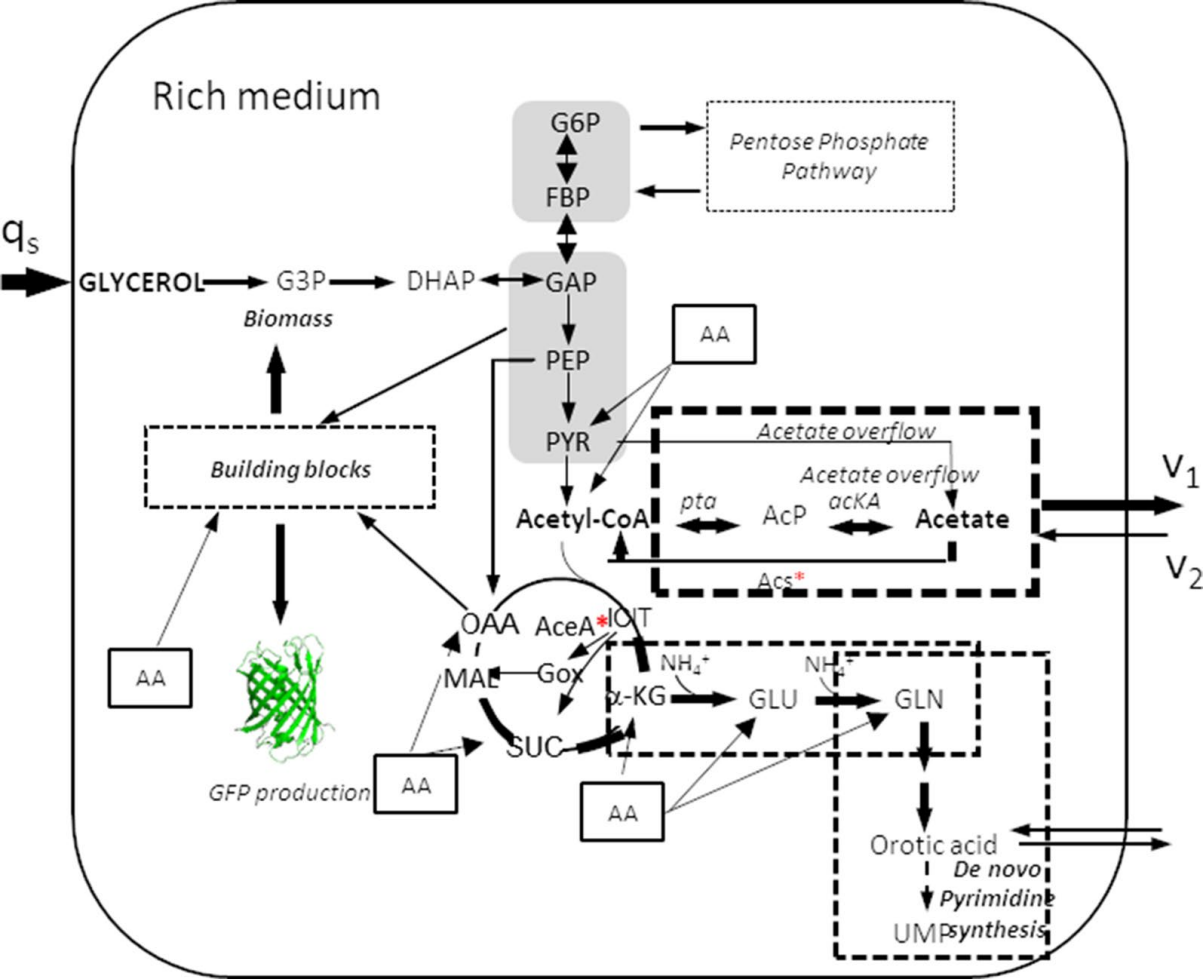
Simplified model for the central metabolic network of the Δ*cobB* strain growing on glycerol-TB7. The red asterisk represents protein acetylation. (q_s_, data shown in Table 2; v_1_, acetate production rate and v_2_, acetate consumption rate, data shown in Table 3)

CobB is the best characterized deacetylase in *E. coli*, although other *E. coli* proteins such as acetyl-CoA synthetase (Acs) and phosphotransacetylase (Pta) have been reported to functionally act as deacetylases [60, 61]. In this study, the *cobB* deletion strain exhibited a physiology similar to the wt strain (Tables 1 and 2). However, Δ*cobB* showed a 2.5-fold decrease relative to the wt in the assimilation of acetate, and it was similar to that of the *acs* mutant (Fig. 2 and Table 3). In *E. coli*, Acs activity is regulated by CobB-mediated deacetylation [56, 62] (Fig. 1). As a result, the deletion of the sirtuin enzyme would involve an increase in the Acs lysine acetylation level, which could induce a total or partial inhibition of this enzyme and a consequent block of acetate incorporation by the Acs pathway that was partially offset by the Pta–AckA pathway. However, in the Δ*cobB* strain, the production of GFP was increased 1.3- to 2-fold with respect to wt with more relevance in glycerol-TB7 (Figs. 5, 6, 7). In addition to that fact, extracellular orotic acid was detected at very low concentrations in *E. coli* BL21 wt and its mutant derivatives in all media, except in the Δ*cobB* mutant. This mutant showed between 0.12 and 0.72 mM, well above the 0.05 mM threshold obtained in the rest of the strains, although this extracellular orotate concentration would not involve too much loss of carbon flux for cell growth. This metabolite allows a 3-way interconnection: de novo pyrimidine synthesis, ammonia assimilation and the TCA cycle through α-KG (Fig. 10). It has been shown that CobB controls acetylation of isocitrate lyase [56], and thus, the glyoxylate shunt might be affected by the increased acetylation in the Δ*cobB* mutant. In this situation, flux through the glyoxylate shunt decreased and increased the flux through the TCA cycle resulting in an increase in the α-KG concentration and finally in the increase in orotic concentration. Moreover, it has also been shown that glyoxylate shunt proteins are less abundant in the Δ*cobB* mutant [56, 63]. The isocitrate node is critical in controlling the ratio of carbon destined for cell growth and energy production, which could have an impact on protein production. All of these observations were additionally aggravated through the availability of free amino acids (Fig. 10). These elevated values of GFP expression could also be due to the importance of lysine acetylation as a modification that can regulate the activity of some proteins involved in the process of gene transcription. Thereby, it must trigger an increase in protein production because it has been reported that CobB-regulated acetylation sites are related to transcription, translation and DNA binding [57]. In accordance with these results, a recently published work suggested that *E. coli* Δ*cobB* can produce higher levels of recombinant proteins, as well as cope with cell stress derived from the production of recombinant proteins better than wt strain [28]. Furthermore, GFP acetylation levels observed in the Δ*cobB* strain were similar to the wt in complex media cultures, but acetylation levels in the Δ*cobB* strain were higher than the wt in minimal media. This result suggests a more relevant role of this deacetylase in defined media, which could be due to a higher control over acetate metabolism and non-enzymatic acetylation in these conditions [64]. A simplified model for the central metabolic network of Δ*cobB* strains is shown in Fig. 10.

Acs catalyses a high-affinity acetate incorporation pathway in *E. coli*. In this study, we observed that the *acs* deletion strain showed slightly less GFP expression than the wt strain in MM9, although these results were not statically significant (Fig. 6). In TB7 media, *acs* deletion decreased the efficiency of acetate reincorporation, but GFP production was not substantially affected. Δ*acs* exhibited elevated µ_max_ and Y_X/S_ values, and these values were comparable to the wt strain (Tables 1 and 2). Some studies carried out in *E. coli* K12 showed no differences in µ_max_ between the wt strain and Δ*acs* mutant [50, 65], even though *acs* is expressed more in the B strain and its deletion seems to have no effect on the growth rate. It was shown that *E. coli* activation of carbon catabolite repression and repression of *acs* take place simultaneously prior to the start of overflow metabolism [65], and recently, it has been shown that the Pta–AckA pathway was found to be essential for both flux directions, while alternative routes (Acs or PoxB) play virtually no role in glucose consumption [25]. In general, the behaviour of the Δ*acs* strain was similar to that of the wild-type, and the level of acetylation of GFP was almost the same as wt.

**Fig. 11:**
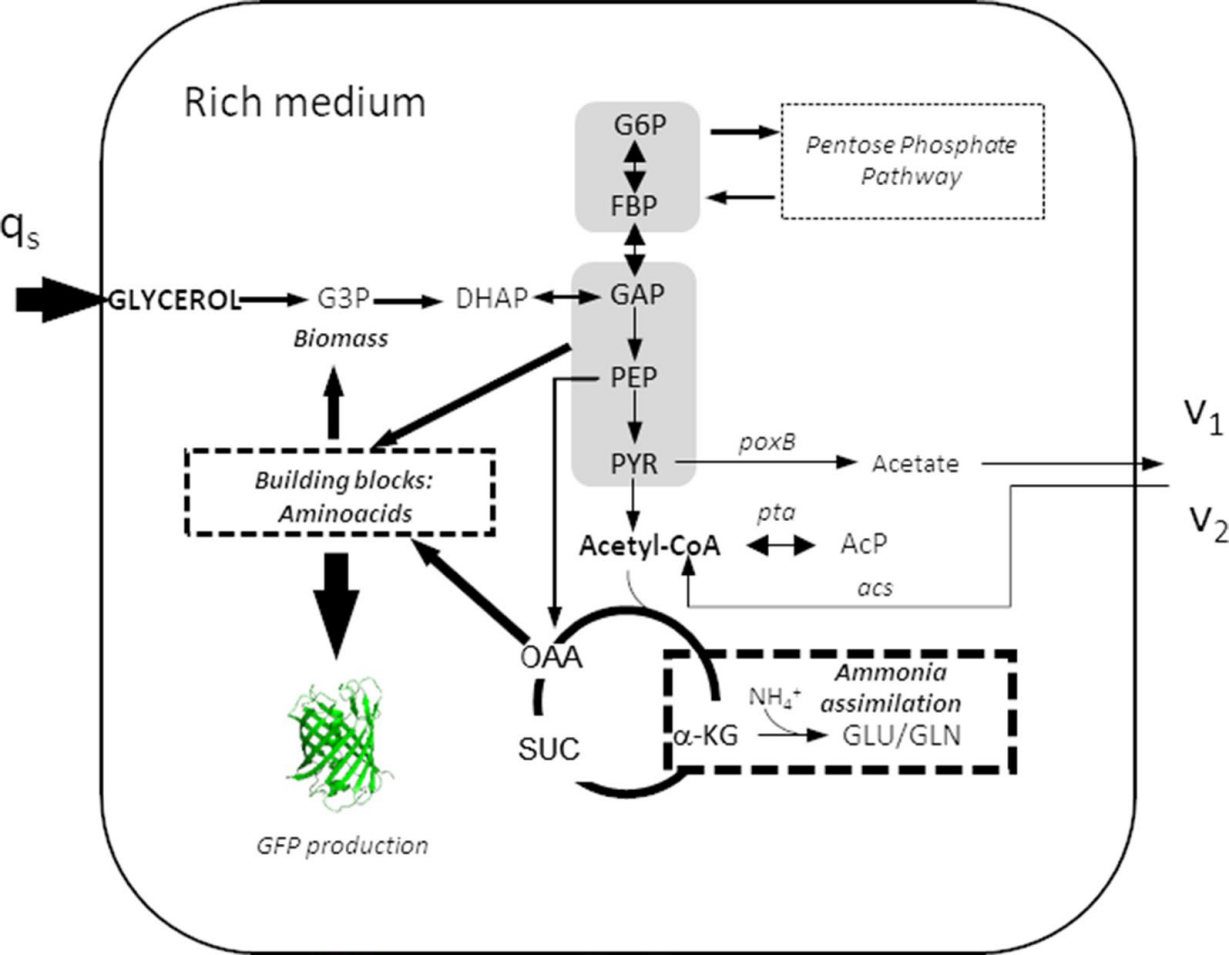
Simplified model of the central metabolic network of the Δ*ackA* strain growing on glycerol-TB7. (q_s_, data shown in Table 2; v_1_, acetate production rate and v_2_, acetate consumption rate, data shown in Table 3)

The deletion of the Pta–AckA pathway revealed major differences with respect to the wt in all parameters evaluated in this study. The Δ*ackA* strain showed the highest GFP expression in all growth media types used (Fig. 6) and increased up to 5.5-fold in glucose-MM9 media when compared to the wt. The observed differences were statistically significant in all the cases with a p-value < 0.0001. GFP expression in the Δ*pta* strain was also increased 1.4- to 1.7-fold with respect to the wt in complex medium (Fig. 6). These strains showed the lowest values of extracellular acetate (Fig. 3a), which was expected since this pathway deletion has been widely employed as a mechanism to overcome acetate accumulation [4, 46, 47, 50]. In glycerol-TB7, the extracellular acetate detected could be formed by PoxB showing a 4.5-fold decrease in the acetate production rate with respect to the wt that used the Pta–AckA pathway in addition to PoxB. It has been shown that the growth of *E. coli* on glycerol could activate some important gluconeogenic genes such as *poxB*, which is involved in acetate recycling [35] or the operation of biosynthetic pathways in which acetate is a metabolic by-product [65]. These mutants showed about a 2.8-fold decrease in the acetate uptake rate via Acs when compared to the wt (Table 3). The essential role carried out by these enzymes in *E. coli* overflow acetate metabolism was demonstrated by the great differences observed in the growth rate [22, 46, 47] (Table 1). The *pta* and *ackA* deletions limited the specific carbon consumption rate, and the carbon source was not consumed completely during the exponential growth phase on TB7 cultures, especially when this medium was supplemented with glucose (Additional file 1: Figures. S5, S6). However, the Y_X/S_ values in *ackA* deletion strain were higher than wt in all culture conditions except for glycerol-MM9, whereas in the *pta* mutant, biomass yield was less than that in the wt (Table 2). These mutants also increased approximately 2 h adaptation time for growing in all media as compared to the parental strain, decrease the acetate production, and Δ*ackA* strain could accumulate acetyl-P, which is a high-energy compound that acts as a phosphoryl donor. These results indicated that, although Δ*ackA* presented difficulties in growing in peptide-based media, it exhibited an adequate distribution of flows by a noticeable decrease in the rate of uptake of carbon sources with a negligible loss of carbon in favour of maximal protein production (Figs. 4 and 11).

Two previous works improved protein production through *pta* and *ackA* deletion. Thus, in 2003, Kim et al. employed an antisense strategy to decrease *pta* and *ackA* expression, increasing GFP expression between 1.6- and 2-fold with respect to the wt strain [66]. In the second study, a double mutant *pta*–*ackA* produced 1.56-fold more protein than the wt strain [67]. Our data showed the highest protein expression reported to date, obtaining a 3.9- to 5-fold increase with respect to the wt using the Δ*ackA* strain (Fig. 6).

On the other hand, in all types of growth media tested, the highest protein acetylation level corresponded to GFP purified from the Δ*ackA* strain (Fig. 8). This elevated level of acetylation was due to acetyl-P accumulation (Fig. 1), which acts as an acetyl donor in chemical acetylation. In contrast, the lowest acetylation level was observed for GFP purified from the *pta* deletion strain. This low level of lysine acetylation could be explained by the block in the acetyl-P production in this mutant strain (Fig. 1). These two acetylation profiles have been previously reported [57, 58]. The accumulation of acetyl-P could lead to non-enzymatic acetylation and, subsequently, could affect the regulation of metabolic processes. Therefore is an aspect to take into account this mutant is employ. Furthermore, GFP purified from the Δ*ackA* strain showed the same native conformation as purified GFP from the rest of the conditions tested without a relationship observed between the lysine acetylation level and native protein conformation.

## Conclusions

The objective of this study was to improve the yield of recombinant proteins produced by bioprocesses based in *E. coli* BL21 metabolism, tackling different strategies simultaneously. Thus, the use of diverse carbon and nitrogen sources and acetate metabolism knockout strains can redirect carbon fluxes to different pathways and affect the final yield of the bioprocess. We concluded that the use of TB7 complex medium and glycerol as a carbon source involves an increase in protein production using *E. coli* BL21 wt or its mutant derivatives. Moreover, *ΔackA* strain *E. coli* produced an increase, up to fivefold, in protein production with respect to the wt strain, and it exhibited an adequate distribution of flows since a negligible extracellular acetate concentration and loss of carbon was detected for this knockout strain, which favours maximal protein production. Finally, the acetylation level of the protein produced must be taken into account, as it is altered by culture medium and metabolic fluxes.

## Methods

### Plasmids and strains

*Escherichia coli* strains, plasmids and primers used in this study are listed in Additional file 1: Tables S1 and S2. *E. coli* BL21(DE3) was purchased from Sigma-Aldrich, and its knockout strains were constructed using the phage lambda Red recombinase method [68]. pRSETA–EmGFP overexpression plasmid (whose recombinant expression gene is controlled by the T7 promoter) was purchased from Invitrogen.

### Growth analysis and HPLC analysis of metabolites

Chemically competent *E. coli* BL21 (DE3) wt and knockout strains were transformed by heat shock at 42 °C with the pRSETA-GFP plasmid and grown in batch mode in minimal M9 medium (10 mM (NH_4_)_2_SO_4_, 8.5 mM NaCl, 40 mM Na_2_HPO_4_, 20 mM KH_2_PO_4_, 185 μM FeCl_3_, 175 μM EDTA, 7 μM ZnSO_4_, 7 μM CuSO_4_ · 5 H_2_O, 7 μM MnSO_4_ , 7 μM CoCl_2_ , 1 mM MgSO_4_, 0.1 mM CaCl_2_ and 1 μM thiamine · HCl) or in complex TB7 medium (10 g/L tryptone buffered at pH 7.0 with 100 mM potassium phosphate) supplemented with glucose (20 mM) or glycerol (40 mM) as the carbon sources. Cultures were inoculated with an initial optical density (OD_600_) of 0.05 units with exponentially growing precultures and induced with 0.1 mM IPTG at 0.5 OD_600_. The specific growth rate was determined [69], and kinetic and stoichiometric parameters were calculated as indicated by Martinez-Gómez et al. [35].

To quantify extracellular metabolites, 1 mL culture samples were taken at different culture phases and cells were harvested by centrifugation. Extracellular metabolites were analysed by an HPLC equipped with a differential refractometer and UV detectors (Shimadzu Scientific Instruments) using an ion exclusion column (ICSep Coregel 87H3, Transgenomic). The mobile phase was 5 mM H_2_SO_4_ flowing at 0.5 mL/min and 65 °C. Glucose consumption was determined by the dinitrosalicylic acid (DNS) method [70]. Statistical test was performed using Prism v7 (GraphPad) analytical software.

### Quantitative real-time PCR (qRT-PCR)

*Escherichia coli* BL21 wt strain was grown in batch mode in minimal M9 medium supplemented with glucose (20 mM) or glycerol (40 mM) as carbon sources. Cells were collected by centrifugation at the exponential phase, when OD_600_ reached 1, and the pellet was frozen at − 80 °C until RNA extraction. Total RNA was purified using Vantage™ Total RNA Purification Kit (Origene) with the manufacturer’s protocol. Residual DNA was removed by RNase-Free DNase Set (Qiagen), and the concentration and purity of RNA was determined using a Nano-Drop 2000 spectrophotometer (Thermo Scientific). cDNAs were synthetized from 1 µg of RNA using TaqMan™ Reverse Transcription Reagents (Applied Biosystems) according to the manufacturer’s protocol. qRT-PCR experiments were carried out on the QuantStudioTM 5 Flex (Applied Biosystems) using Power SYBR™ Green PCR Master Mix (Invitrogen) according to the manufacturer´s protocol. Amplification conditions were 2 min at 50 °C then 10 min at 95 °C, followed by 40 cycles of 2 steps at 95 °C for 15 s and 60 °C for 1 min, and a final dissociation cycle of 95 °C for 15 s, 60 °C for 1 min and 95 °C for 15 s. Primers sequences used in this study are listed in Additional file 1: Table S2. *rrs* of the 16S rRNA gene was chosen as internal control to normalize the data, and 2^−ΔΔCT^ method was employed to analyse the data [71]. Relative gene transcription values of *E. coli* BL21 wt growing in glycerol as the sole carbon source are expressed as compared to the values of the same strain growing in glucose as the sole carbon source. All experiments were performed from at least 3 differents cDNAs from each growth condition, and a triplicate of each gene studied was carried out.

### Overexpression analysis

Chemically competent *E. coli* BL21 (DE3) wt and knockout strains transformed with the pRSETA-GFP plasmid were grown in a microplate reader (Synergy H1 Hybrid Multi-Mode Reader), simultaneously monitoring growth at OD_600_ and fluorescence at 487 nm excitation and 509 nm emission wavelengths. Ninety-six-well plates were sterilized and prepared with 200 μL of minimal M9 or complex TB7 medium supplemented with glucose (20 mM) or glycerol (40 mM) as the sole carbon source. Cultures were inoculated to an initial optical density (OD_600_) of 0.05 units with exponentially growing precultures and induced with 0.1 mM IPTG at 0.5 OD_600_. The 96-well plates were covered with an adhesive gas-permeable sheet (Sigma-Aldrich) to prevent evaporation and permit aeration. Cultures were grown in quintuplicate with double orbital shaking at 37 °C for 24 h. Fluorescence counts were recorded during growth, and the maximum fluorescence values of GFP expression, corresponding to the stationary growth phase, were normalized to the OD_600_ values (relative fluorescence), and the resulting data were expressed as the expression level relative to that obtained for wt. Statistical test was performed using Prism v7 (GraphPad) analytical software.

### Protein purification

Chemically competent *E. coli* BL21 (DE3) wt and knockout strains were transformed by heat shock at 42 °C with the expression pRSETA-GFP plasmid. Cultures were grown overnight in batch mode at 30 °C with orbital shaking (250 rpm). The culture medium used was minimal M9 or complex TB7 medium supplemented with glucose (20 mM) or glycerol (40 mM) as the sole carbon source. Cultures were inoculated to an initial optical density (OD_600_) of 0.05 units from exponentially growing precultures and induced with 0.1 mM IPTG at 0.5 OD_600_. Cell pellets were harvested by centrifugation (20 min; 4500×*g*) and resuspended in binding buffer (50 mM potassium phosphate, 500 mM NaCl, 25 mM imidazole, pH 8). Cells were disrupted by sonication for 2 min (20 s each pulse) using a Vibra Cell sonicator (Sonicator Sonics & Materials). The lysates were clarified by centrifugation at 14,000×*g* for 30 min at 4 °C. The supernatants were applied onto a Ni(II)-loaded 5 mL His-Trap HP column (GE Heathcare) previously equilibrated in binding buffer. Proteins were eluted using a linear gradient of imidazole from 0 to 500 mM at a flow rate of 5 mL/min. The protein buffer was then changed to buffer A (50 mM potassium phosphate, pH 7.5) using a HiPrep™ 26/10 desalting column (GE Heathcare) at a flow rate of 9 mL/min.

### Western immunoblot analysis

The GFP acetylation level was studied by western blot immunoassay. The purified protein concentration was normalized after the amount of protein was determined. Samples were separated on 10% acrylamide SDS-PAGE and transferred to polyvinylidene fluoride (PVDF) membranes using a semidry transfer unit (Trans-Blot^®^ SD Semi-Dry Transfer Cell, Bio-Rad). The membranes were blocked with 1% (w/v) bovine serum albumin (BSA) in TBST (50 mM Tris–HCl, 150 mM NaCl, 0.05% Tween-20, pH 7.5) for 1 h. The blot was then incubated with primary rabbit monoclonal anti-acetyl Lys antibody (InmuneChem) at a 1:2000 dilution overnight at 4 °C. The membrane was washed 3 times for 10 min each with TBST and then incubated with HRP-conjugated goat anti-rabbit secondary antibody (Santa Cruz Biotechnology) at a 1:15,000 dilution for 1 h at room temperature. The blot was washed 6 times for 5 min each time with TBST. Finally, the membrane was incubated for 10 min with SuperSignal™ West Pico Chemiluminescent Substrate (Thermo Scientific) and revealed with a chemiluminescence Amersham Imager 600 (GE Healthcare). ImageJ Gel Analyzer software was used for densitometric quantification. A stripping protocol was carried out, and the membranes were incubated with primary mouse monoclonal anti-Xpress at a 1:3000 dilution (Invitrogen) as a loading control, while the secondary antibody was an HRP-conjugated goat anti-mouse secondary antibody (Invitrogen) at a 1:15,000 dilution.

## Supplementary information

**Additional file 1: Table S1.** List of plasmid used in this study. **Table S2.** List of primers employed in this study. **Figure S1.** *E. coli* BL21 wt grown at OD_600_ and acetate, glucose and glycerol extracellular concentrations. *E. coli* BL21 wt growth (black), extracellular acetate (blue) and glucose or glycerol consumption (green) in TB7 glucose (A), TB7 glycerol (B), MM9 glucose (C) and MM9 glycerol (D). **Figure S2.** *E. coli* BL21 ΔpatZ grown at OD_600_ and acetate, glucose and glycerol extracellular concentrations. *E. coli* BL21 ΔpatZ growth (black), extracellular acetate (blue) and glucose or glycerol consumption (green) in TB7 glucose (A), TB7 glycerol (B), MM9 glucose (C) and MM9 glycerol (D). **Figure S3.** *E. coli* BL21 ΔcobB grown at OD_600_ and acetate, glucose and glycerol extracellular concentrations. *E. coli* BL21 ΔcobB growth (black), extracellular acetate (blue) and glucose or glycerol consumption (green) in TB7 glucose (A), TB7 glycerol (B), MM9 glucose (C) and MM9 glycerol (D). **Figure S4.** *E. coli* BL21 Δacs grown at OD_600_ and acetate, glucose and glycerol extracellular concentrations. *E. coli* BL21 Δacs growth (black), extracellular acetate (blue) and glucose or glycerol consumption (green) in TB7 glucose (A), TB7 glycerol (B), MM9 glucose (C) and MM9 glycerol (D). **Figure S5.** *E. coli* BL21 ΔackA grown at OD_600_ and acetate, glucose and glycerol extracellular concentrations. *E. coli* BL21 ΔackA growth (black), extracellular acetate (blue) and glucose or glycerol consumption (green) in TB7 glucose (A), TB7 glycerol (B), MM9 glucose (C) and MM9 glycerol (D). **Figure S6.** *E. coli* BL21 Δpta grown at OD_600_ and acetate, glucose and glycerol extracellular concentrations. *E. coli* BL21 Δpta growth (black), extracellular acetate (blue) and glucose or glycerol consumption (green) in TB7 glucose (A), TB7 glycerol (B), MM9 glucose (C) and MM9 glycerol (D). **Figure S7.** Orotate extracellular concentration. Orotate extracellular concentration detected for *E. coli* BL21 wt, Δ*patZ*, Δ*cobB*, Δ*acs*, Δ*ackA* and Δ*pta* growing in TB7 glucose (A), TB7 glycerol (B), MM9 glucose (C) and MM9 glycerol (D). **Figure S8.** GFP fluorescence images. GFP fluorescence images of plating of *E. coli* BL21 wt (1) and mutant derivative (2: Δ*patZ*; 3: Δ*cobB*; 4: Δ*acs*; 5: Δ*ackA*; 6: Δ*pta*) cells expressing GFP. (A): plate image. (B): fluorescence plate image obtained with the Cy2 filter allowing GFP fluorescence detection with the Amersham Imager 600 (GE Healthcare). **Figure S9.** Relative fluorescence recorded during culture growth of E. coli BL21 wt in glycerol-TB7.

## Data Availability

All data generated or analyzed during this study are included in this published article.
