# Supplementary material for: Engineering protein production by rationally choosing a carbon and nitrogen source using E. coli BL21 acetate metabolism knockout strains
Source: Microb Cell Fact. 2019 Sep 4;18:151. doi: 10.1186/s12934-019-1202-1 (PMC6724240; doi:10.1186/s12934-019-1202-1)
Supplement: Supplementary file 1 — Additional file 1: Table S1. List of plasmid used in this study. Table S2. List of primers employed in this study. Figure S1. E. coli BL21 wt grown at OD600 and acetate, glucose and glycerol extracellular concentrations. E. coli BL21 wt growth (black), extracellular acetate (blue) and glucose or glycerol consumption (green) in TB7 glucose (A), TB7 glycerol (B), MM9 glucose (C) and MM9 glycerol (D). Figure S2. E. coli BL21 ΔpatZ grown at OD600 and acetate, glucose and glycerol extracellular concentrations. E. coli BL21 ΔpatZ growth (black), extracellular acetate (blue) and glucose or glycerol consumption (green) in TB7 glucose (A), TB7 glycerol (B), MM9 glucose (C) and MM9 glycerol (D). Figure S3. E. coli BL21 ΔcobB grown at OD600 and acetate, glucose and glycerol extracellular concentrations. E. coli BL21 ΔcobB growth (black), extracellular acetate (blue) and glucose or glycerol consumption (green) in TB7 glucose (A), TB7 glycerol (B), MM9 glucose (C) and MM9 glycerol (D). Figure S4. E. coli BL21 Δacs grown at OD600 and acetate, glucose and glycerol extracellular concentrations. E. coli BL21 Δacs growth (black), extracellular acetate (blue) and glucose or glycerol consumption (green) in TB7 glucose (A), TB7 glycerol (B), MM9 glucose (C) and MM9 glycerol (D). Figure S5. E. coli BL21 ΔackA grown at OD600 and acetate, glucose and glycerol extracellular concentrations. E. coli BL21 ΔackA growth (black), extracellular acetate (blue) and glucose or glycerol consumption (green) in TB7 glucose (A), TB7 glycerol (B), MM9 glucose (C) and MM9 glycerol (D). Figure S6. E. coli BL21 Δpta grown at OD600 and acetate, glucose and glycerol extracellular concentrations. E. coli BL21 Δpta growth (black), extracellular acetate (blue) and glucose or glycerol consumption (green) in TB7 glucose (A), TB7 glycerol (B), MM9 glucose (C) and MM9 glycerol (D). Figure S7. Orotate extracellular concentration. Orotate extracellular concentration detected for E. coli BL21 wt, ΔpatZ, ΔcobB, Δacs, Δac [file 12934_2019_1202_MOESM1_ESM.docx]

**Additional file 1**

**Engineering protein production by rationally choosing a carbon and nitrogen source using *E. coli* BL21 acetate metabolism knockout strains**

**Gema Lozano Terol, Julia Gallego-Jara*, Rosa Alba Sola Martínez, Manuel Cánovas Díaz and Teresa de Diego*.**

**Department of Biochemistry and Molecular Biology (B) and Immunology, Faculty of Chemistry, University of Murcia, Campus of Espinardo, Regional Campus of International Excellence ‘‘Campus Mare Nostrum’’, P.O. Box 4021, Murcia E-30100, Spain.**

***Corresponding author:**

**Email:** [**julia.gallego@um.es**](mailto:julia.gallego@um.es) **and** [**tdp@um.es**](mailto:tdp@um.es)**; Tel.: +34 868887397; Fax: +34 868 88 4148.**

**Tables**

**Table S1:** **list of plasmid used in this study.**

| Plasmid | Description | Source |
| --- | --- | --- |
| pKD13 | Amp^R^/Kam^R^, resistance cassette flanked by FRT regions. | Yale *E. coli* Genetic Stock Center |
| pRSETA-EmGFP | Amp^R^, pRSETA with GFP gene. T7 promoter. | Invitrogen |
| pKD46 | Amp^R^, this plasmid is temperature sensitive replication at 30°C and arabinose inducible Red recombinase expression. | Yale *E. coli* Genetic Stock Center |

**Table S2: list of primers employed in this study.**

| Primer | Sequence 5´🡪 3´ |
| --- | --- |
| Primers used for knockout strains constructions | |
| *patZ* KO Fwd | GTTAGAAAGCGTTTAAAATCATTCGGTCACTTCTGCGGGAGACCGGTATGATTCCGGGGATCCGTCGACC |
| *patZ* KO Rev | TTAAGTGGTCAACATTTCCAGTACCTTACTCATGATTCCTCGCGCTGG GCTGTAGGCTGGAGCTGCTTCG |
| *cobB* KO Fwd | GTGGTGCGGCCTTCCTACATCTAACCGATTAAACAACAGAGGTTGCTATGATTCCGGGGATCCGTCGACC |
| *cobB* KO Rev | CCCCTTGCAGGCCTGATAAGCGTAGTGCATCAGGCAATGCTTCCCGCTTTTGTAGGCTGGAGCTGCTTCG |
| *acs* KO Fwd | AACGCTTATGCCACATATTATTAACATCCTACAAGGAGAACAAAAGCATGATTCCGGGGATCCGTCGACC |
| *acs* KO Rev | GTTACCGACTCGCATCCGGCAATTGTGGGTTACGATGGCATCGCGATAGCTGTAGGCTGGAGCTGCTTCG |
| *ackA* KO Fwd | TGGCTCCCTGACGTTTTTTTAGCCACGTATCAATTATAGGTACTTCCATGATTCCGGGGATCCGTCGACC |
| *ackA* KO Rev | GCACCGCCAGCTGAGCTGGCGGTGTGAAATCAGGCAGTCAGGCGGCTCGCTGTAGGCTGGAGCTGCTTCG |
| *pta* KO Fwd | GCTGTTTTGTAACCCGCCAAATCGGCGGTAACGAAAGAGGATAAACCGTGATTCCGGGGATCCGTCGACC |
| *pta* KO Rev | GCAGCGCAAAGCTGCGGATGATGACGAGATTACTGCTGCTGTGCAGACTGTGTAGGCTGGAGCTGCTTCG |
| *patZ* Check Fwd | GCTATCTGGCAGGAAAAACG |
| *patZ* Check Rev | GCAGACGACATAAGCGGGCA |
| *cobB* Check Fwd | TGTTCCGCGCATTGAACGC |
| *cobB* Check Rev | AACGCCTTATCCGGCCCAC |
| *acs* Check Fwd | GCGTGATCTGTCGCCCAAAT |
| *acs* Check Rev | AATGCCTGATGCGACGCTGT |
| *ackA* Check Fwd | GCAGTGCATGATGTTAATC |
| *ackA* Check Rev | TTATCCTCTTTCGTTACCG |
| *pta* Check Fwd | AACGAAGAACTGGTTATCGC |
| *pta* Check Rev | AGTGATTATTTCCGGTTCAG |
| K1 | CAGTCATAGCCGAATAGCCT |
| K2 | CGGTGCCCTGAATGAACTGC |
| Primers used for RT-qPCR assays | |
| *acs* Fwd | ACACACCATTCCTGCCAACA |
| *acs* Rev | TGTTGATACATCGCCTCGTACTG |
| *aceA* Fwd | TGCACGGTGAGTCGAAAAAA |
| *aceA* Rev | TAGACTGCTTCAATACCCGCTTT |
| *ppsA* Fwd | CCTCCCTGGGTGAAATGATTA |
| *ppsA* Rev | GGCGGTTGTGGCGAAA |
| *glpK* Fwd | GTGGCGCAGTAGCAAACAATT |
| *glpK* Rev | ACTTCCGGGCGCTCAAC |
| *acnB* Fwd | GGCGAGAAGATCGACGAAGT |
| *acnB* Rev | GCACGGAAGTGACCGATGTT |
| *aceE* Fwd | CGTGAAGAAGGTGTTGAGCG |
| *aceE* Rev | TTGCTGATACCTGTGCCTGC |
| *gapA* Fwd | GAAAGGCGTTCTGGGCTACA |
| *gapA* Rev | GCAAACTTCGCCGTTGAAA |
| *tpiA* Fwd | AACTCCGGCTCAGGCACAG |
| *tpiA* Rev | AGCCGCCGTACTGAATGATC |
| *rrsA* Fwd | CCTTACGACCAGGGCTACACA |
| *rrsA* Rev | CACTTTATGAGGTCCGCTTGCT |

**Figures**

**Figure S1** **E. coli BL21 wt grown at OD_600_ and acetate, glucose and glycerol extracellular concentrations.** E. coli BL21 wt growth (black), extracellular acetate (blue) and glucose or glycerol consumption (green) in TB7 glucose (A), TB7 glycerol (B), MM9 glucose (C) and MM9 glycerol (D).

**Figure S2** **E. coli BL21 ΔpatZ grown at OD_600_ and acetate, glucose and glycerol extracellular concentrations.** E. coli BL21 ΔpatZ growth (black), extracellular acetate (blue) and glucose or glycerol consumption (green) in TB7 glucose (A), TB7 glycerol (B), MM9 glucose (C) and MM9 glycerol (D).

**Figure S3** **E. coli BL21 ΔcobB grown at OD_600_ and acetate, glucose and glycerol extracellular concentrations.** E. coli BL21 ΔcobB growth (black), extracellular acetate (blue) and glucose or glycerol consumption (green) in TB7 glucose (A), TB7 glycerol (B), MM9 glucose (C) and MM9 glycerol (D).

**Figure S4** **E. coli BL21 Δacs grown at OD_600_ and acetate, glucose and glycerol extracellular concentrations.** E. coli BL21 Δacs growth (black), extracellular acetate (blue) and glucose or glycerol consumption (green) in TB7 glucose (A), TB7 glycerol (B), MM9 glucose (C) and MM9 glycerol (D).

**Figure S5** **E. coli BL21 ΔackA grown at OD_600_ and acetate, glucose and glycerol extracellular concentrations.** E. coli BL21 ΔackA growth (black), extracellular acetate (blue) and glucose or glycerol consumption (green) in TB7 glucose (A), TB7 glycerol (B), MM9 glucose (C) and MM9 glycerol (D).

**Figure S6** **E. coli BL21 Δpta grown at OD_600_ and acetate, glucose and glycerol extracellular concentrations.** E. coli BL21 Δpta growth (black), extracellular acetate (blue) and glucose or glycerol consumption (green) in TB7 glucose (A), TB7 glycerol (B), MM9 glucose (C) and MM9 glycerol (D).

**Figure S7** **Orotate extracellular concentration.** Orotate extracellular concentration detected for *E. coli* BL21 wt, Δ*patZ*, Δ*cobB*, Δ*acs*, Δ*ackA* and Δ*pta* growing in TB7 glucose (A), TB7 glycerol (B), MM9 glucose (C) and MM9 glycerol (D).


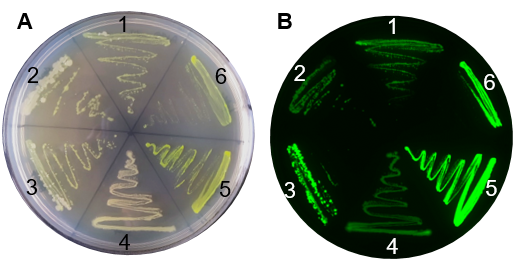


**Figure S8** **GFP fluorescence images.** GFP fluorescence images of plating of *E. coli* BL21 wt (1) and mutant derivative (2: Δ*patZ*; 3: Δ*cobB*; 4: Δ*acs*; 5: Δ*ackA*; 6: Δ*pta*) cells expressing GFP. (A): plate image. (B): fluorescence plate image obtained with the Cy2 filter allowing GFP fluorescence detection with the Amersham Imager 600 (GE Healthcare).


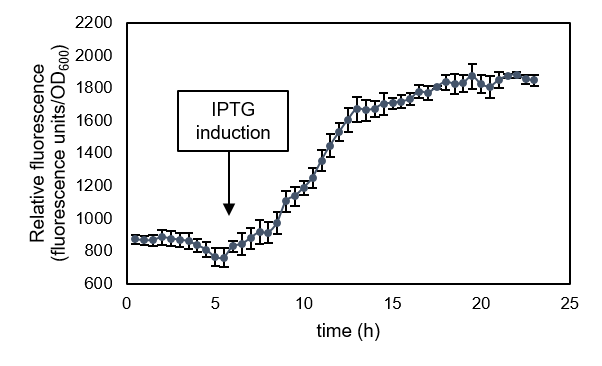


**Figure S9 Relative fluorescence recorded during culture growth of E. coli BL21 wt in glycerol-TB7.**
